# High levels of floridoside at high salinity link osmoadaptation with bleaching susceptibility in the cnidarian-algal endosymbiosis

**DOI:** 10.1242/bio.045591

**Published:** 2019-12-16

**Authors:** Hagen M. Gegner, Nils Rädecker, Michael Ochsenkühn, Marcelle M. Barreto, Maren Ziegler, Jessica Reichert, Patrick Schubert, Thomas Wilke, Christian R. Voolstra

**Affiliations:** 1 Red Sea Research Center, Division of Biological and Environmental Science and Engineering (BESE), King Abdullah University of Science and Technology (KAUST), Thuwal 23955, Saudi Arabia; 2 Division of Science and Engineering, New York University Abu Dhabi (NYUAD), Saadiyat Island, Abu Dhabi, United Arab Emirates; 3 Department of Animal Ecology & Systematics, Justus Liebig University, 35390 Giessen, Germany; 4 Department of Biology, University of Konstanz, 78457 Konstanz, Germany

**Keywords:** Coral reefs, Coral bleaching, Aiptasia, Climate change, Symbiosis, Symbiodiniaceae

## Abstract

Coral reefs are in global decline mainly due to increasing sea surface temperatures triggering coral bleaching. Recently, high salinity has been linked to increased thermotolerance and decreased bleaching in the sea anemone coral model Aiptasia. However, the underlying processes remain elusive. Using two Aiptasia host­–endosymbiont pairings, we induced bleaching at different salinities and show reduced reactive oxygen species (ROS) release at high salinities, suggesting a role of osmoadaptation in increased thermotolerance. A subsequent screening of osmolytes revealed that this effect was only observed in algal endosymbionts that produce 2-O-glycerol-α-D-galactopyranoside (floridoside), an osmolyte capable of scavenging ROS. This result argues for a mechanistic link between osmoadaptation and thermotolerance, mediated by ROS-scavenging osmolytes (e.g., floridoside). This sheds new light on the putative mechanisms underlying the remarkable thermotolerance of corals from water bodies with high salinity such as the Red Sea or Persian/Arabian Gulf and holds implications for coral thermotolerance under climate change.

This article has an associated First Person interview with the first author of the paper.

## Abstract (precis)

**Summary:** Using the coral model Aiptasia, we show increased thermotolerance at high salinities, concomitant with a reduced reactive oxygen species (ROS) release by algal endosymbionts. This suggests a mechanistic link between osmoadaptation and thermotolerance, mediated by ROS-scavenging osmolytes.

## INTRODUCTION

Climate change leads to ocean warming and ocean acidification, which are threatening coral reefs at a global scale ([Bibr BIO045591C15][Bibr BIO045591C15]). While ocean warming is identified as the main driver of coral bleaching (Hughes et al., 2017b), the effects of ocean acidification are less clear, but presumably affect coral calcification and reef growth (Tambutté et al., 2015; Albright et al., 2016; Liew et al., 2018). Bleaching describes the loss of the coral-associated micro-algal photosynthetic endosymbionts in the family Symbiodiniaceae ([Bibr BIO045591C16][Bibr BIO045591C16]; LaJeunesse et al., 2018). As such, corals lose their supply of photosynthates, which covers their main energy needs to build and maintain calcium carbonate skeletons that in turn provide the structural foundation of reef ecosystems (Muscatine and Porter, 1977). Hence, it is becoming increasingly important to better understand the mechanisms and drivers of coral bleaching, as well as the factors that determine stress resilience and thermotolerance of corals (Torda et al., 2017).

As a rough estimate, corals start to bleach at about 1–2°C above their annual average summer temperatures (Hoegh-Guldberg, 1999), suggesting that corals are adapted to their prevailing environmental conditions ([Bibr BIO045591C16][Bibr BIO045591C16]), as supported by observed differences in thermotolerance across regions (Osman et al., 2018). To date, we are missing a detailed understanding of the factors that contribute to such geographical differences of bleaching susceptibility. In addition, the cellular mechanisms of bleaching are not completely understood. While the production and accumulation of reactive oxygen species (ROS) as a consequence of increased temperatures, i.e. heat stress, certainly play a role (Lesser, 1997, 2011; Weis, 2008), recent studies have shown bleaching without heat stress (Pogoreutz et al., 2017), bleaching without light (Tolleter et al., 2013), and bleaching decoupled from oxidative stress (Nielsen et al., 2018). Further, Gegner et al. (2017) showed increased thermotolerance and reduced bleaching at high salinities in the coral model Aiptasia, suggesting a possible role of osmoadaptation in stress resilience of symbiotic cnidarians (Ochsenkühn et al., 2017; Osman et al., 2018). However, the underlying mechanism remained elusive.

With regard to the putative importance of salinity in contributing to thermotolerance, it is important to note that some of the most thermotolerant corals can be found in the hottest and most saline water bodies, i.e. the Persian/Arabian Gulf (Hume et al., 2013, 2015; D'Angelo et al., 2015) and the northern Red Sea (Bellworthy and Fine, 2017; Krueger et al., 2017; Osman et al., 2018). To gain insight into the potential mechanisms underlying salinity-conveyed thermotolerance of symbiotic cnidarians, we set up a series of bleaching experiments at different salinities. Using the coral model Aiptasia (*sensu Exaiptasia pallida*), we first assessed the thermotolerance of two host–endosymbiont pairings at different salinity and temperature conditions. Subsequent linking of the heat stress response to ROS and osmolyte levels allowed us to pinpoint potential processes that play a role in the increased thermotolerance and the decreased bleaching at high salinities in Aiptasia.

## RESULTS

### Decreased bleaching concomitant with reduced ROS levels at high salinities suggest a role of osmoadaptation in thermotolerance

**Fig. 1.**
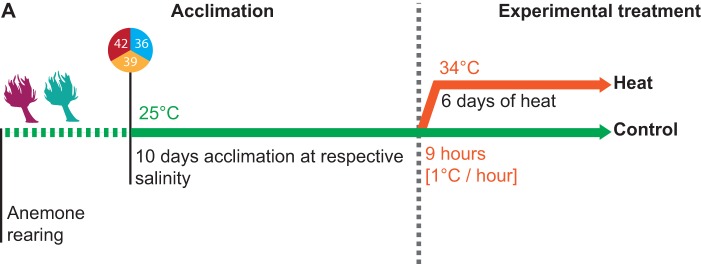
**Overview of experimental procedures.** Two Aiptasia host–endosymbiont pairings (H2-SSB01 and CC7-SSA01) in their symbiotic and aposymbiotic states were acclimated for 10 days to low (36), intermediate (39), and high (42) salinities at ambient temperature, before they were subjected to a 6-day-long heat-stress experiment. A control was kept at ambient temperature throughout the experiment.

To elucidate potential mechanisms underlying salinity-conveyed thermotolerance of symbiotic cnidarians, we assessed physiological and metabolic responses of symbiotic and aposymbiotic anemones of Aiptasia from two host–endosymbiont pairings (H2-SSB01 and CC7-SSA01) under ambient (25°C) and heat stress (34°C) conditions at low (36), intermediate (39), and high (42) salinity (Fig. 1). Briefly, we acclimated anemones to the respective salinities for 10 days. After that, half of the anemones remained at the ambient temperature as a control and the other half was subjected to heat stress for 6 days (see Materials and Methods and Fig. 1).

**Fig. 2.**
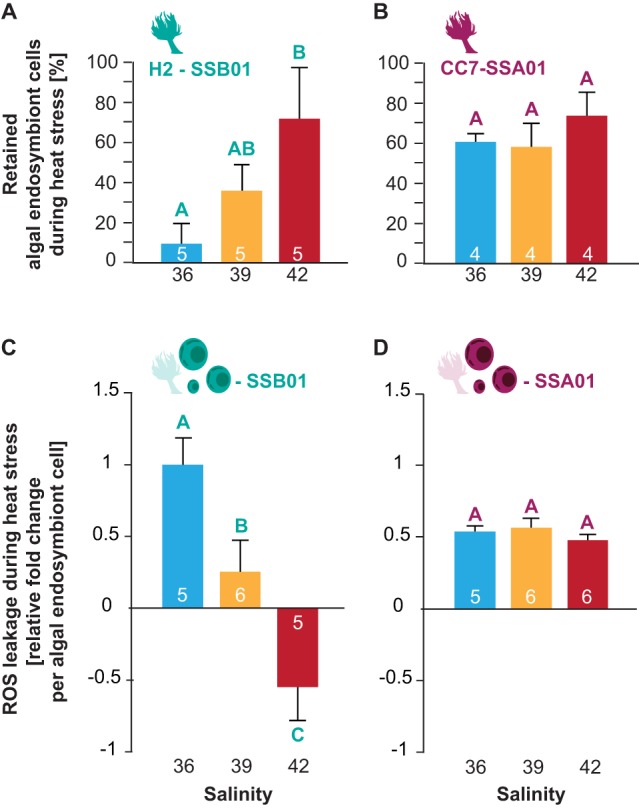
**Effect of different salinities on heat stress-induced bleaching in the coral model Aiptasia.** (A,B) Percentage of retained algal endosymbionts for H2-SSB01 and CC7-SSA01 at low (36), intermediate (39), and high (42) salinities after 6 days of heat stress in relation to control temperatures. (C,D) Relative fold change of ROS leakage per endosymbiont cell for SSB01 and SSA01 at low (36), intermediate (39), and high (42) salinity after 2 days of heat stress in relation to control temperatures. Different letters above bars indicate significant differences between groups (*P*<0.05). Number of replicates is indicated inside the bars. Error bars show the standard error of the mean (s.e.m.).

At ambient temperature (control), salinities had no effect on symbiont densities (ANOVA, H2-SSB01, *F*=2.689, *P*=0.088; CC7-SSA01, *F*=1.467, *P*=0.257; Fig. S1A, Tables S2 and S9) and photosynthetic efficiency (Steel-Dwass, all *P*>0.05; Fig. S1B, Tables S3 and S10) of symbiotic Aiptasia (H2-SSB01 and CC7-SSA01), respectively. Heat stress, on the other hand, caused the loss of endosymbionts in Aiptasia in both host–endosymbiont pairings. In H2-SSB01 the extent of symbiont loss, however, was significantly different at different salinities, with animals at high salinity retaining significantly more algal endosymbionts than animals at low salinity (ANOVA, *F*=5.188, *P*=0.024; Fig. 2A, Table S1A). By comparison, CC7-SSA01 showed overall lower levels of endosymbiont loss compared to H2-SSB01 and retained about the same density of algal endosymbionts independent of salinity (ANOVA, *F*=2.708, *P*=0.120; Fig. 2B; Table S1B). Relative changes in symbiont densities were corroborated by visual bleaching (paling) of anemones (Fig. S1C) and a concomitant decrease of photosynthetic efficiency (Fig. S1B, Table S3). Aposymbiotic anemones, by comparison, did not show any difference in appearance regardless of temperature or salinity (Fig. S1C).

To confirm that decreased bleaching and increased thermotolerance of H2-SSB01 is indeed related to an increase in salinity, we subsequently measured ROS release from freshly isolated algal endosymbionts from symbiotic anemones after 2 days in the respective temperature and salinity treatments. We found that algal endosymbiont types SSB01 and SSA01 (isolated from H2 and CC7, respectively) showed differences in ROS leakage across temperatures and salinities that aligned with the severity of bleaching. While relative ROS leakage decreased for SSB01 at higher salinities (ANOVA, *F*=12.056, *P*<0.001, Fig. 2C; Tables S4 and S11), it was overall lower and unaffected by salinity for SSA01 under heat stress (ANOVA, *F*=0.801, *P*=0.468, Fig. 2D; Tables S4 and S11). Thus, despite the fact that more endosymbionts were retained at high salinity for H2-SSB01 anemones (Fig. 2A), the relative ROS leakage per endosymbiont cell actually decreased during heat stress.

### High levels of the osmolyte floridoside at high salinity concomitant with salinity-conveyed thermotolerance of the Aiptasia holobiont

Given that we found decreased ROS leakage at high salinities, we reasoned that the process of osmoadaptation might be mechanistically linked to the increased thermotolerance at high salinities. Consequently, we screened for osmolytes that are altered in response to increased salinities. One osmolyte that was recently implicated to be pivotal for osmoadaptation of coral holobionts to high-salinity conditions is floridoside, i.e. 2-O-glycerol-α-D-galactopyranoside (Ochsenkühn et al., 2017), produced by the algal endosymbionts. Thus, we characterized carbohydrates and fatty acid metabolites of Aiptasia holobionts, including floridoside among other important osmolytes, antioxidants, and energy carriers.

We identified a total of 34 metabolites from aposymbiotic and symbiotic Aiptasia anemones across temperatures and salinities (Table S12). Metabolic profiles were significantly different between anemone hosts (H2 and CC7) and symbiotic states (H2-SSB01 versus H2-aposymbiotic and CC7-SSA01 versus CC7-aposymbiotic) (Fig. S2; Table S5). Thus, we analyzed metabolic profiles for H2-SSB01 and CC7-SSA01 separately, as our primary interest was on metabolites that were altered in response to salinity and temperature differences in symbiotic cnidarians.

**Fig. 3.**
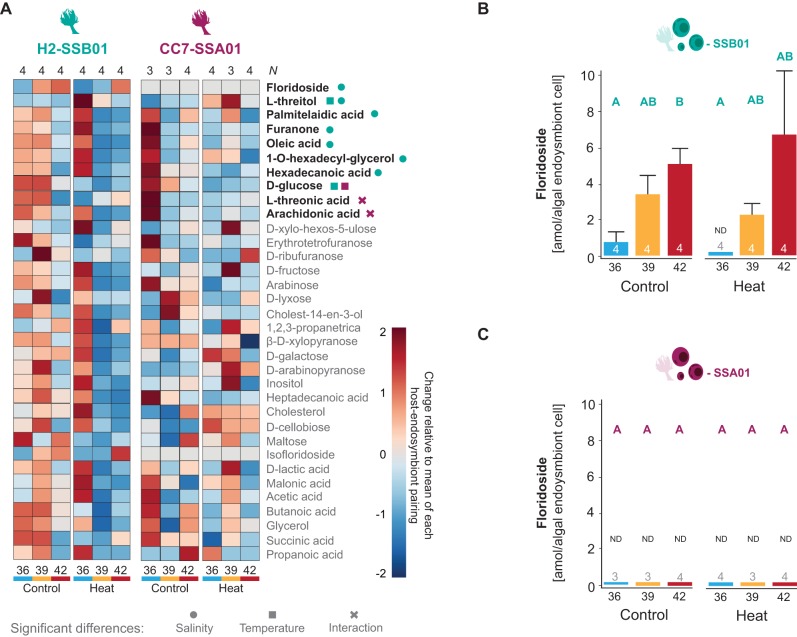
**Metabolite levels of Aiptasia H2-SSB01 and CC7-SSA01 host–endosymbiont pairings.** (A) Heatmaps showing metabolite levels of H2-SSB01 and CC7-SSA01 at ambient (Control) and heat stress (Heat) temperatures at low (36), intermediate (39), and high (42) salinities normalized to total protein content. Significantly different metabolites are designated in bold (two-way ANOVA, all *P*<0.05), with symbols indicating significant differences between salinity (circle), temperature (square), and salinity×temperature interaction (cross). Number of replicates is indicated above the heatmaps (*N*). (B,C) Floridoside levels of H2-SSB01 and CC7-SSA01 at ambient (Control) and heat stress (Heat) temperatures at low (36), intermediate (39), and high (42) salinities per endosymbiont cell. Data were converted from total protein-normalized to algal endosymbiont cell-normalized levels of floridoside using endosymbiont densities as reported in  Fig. S1 (Tables S9 and S13). This is an estimate under the assumption that total protein content largely resembles host protein content. Different letters above bars indicate significant differences between groups (Kruskal–Wallis, *P*<0.05). Number of replicates is indicated above/within bars. ND=not detected. Error bars show the propagated standard error of the mean (s.e.m.).

For H2-SSB01 that increase their thermotolerance at high salinity (see above and Gegner et al., 2017), most metabolites showed decreased levels at high salinity (42) at ambient and heat-stress temperatures (Fig. 3A). In line with this overall pattern, seven metabolites (L-threitol, oleic acid, palmitelaidic acid, 1-O-hexadecylglycerol, hexadecanoic acid, furanone, and floridoside) showed significantly different abundance levels across salinity treatments. Notably, only two metabolites were significantly reduced at heat stress (D-glucose and L-threitol) (two-way ANOVA, *P*<0.05, Fig. 3A; Table S6A), and floridoside was the only measured carbohydrate with increased levels at increasing salinity at ambient and heat-stress temperatures, further confirming its important role as an osmolyte in the cnidarian-dinoflagellate symbiosis (Gegner et al., 2017; Ochsenkühn et al., 2017) (Fig. 3B; Tables S7 and S8).

In comparison to the pronounced metabolic shifts in H2-SSB01, only three metabolites (D-glucose, L-threonic acid, arachidonic acid) were significantly differentially abundant across temperatures and/or salinities in CC7-SSA01 (two-way ANOVA, *P*<0.05, Table S6B). Similar to H2-SSB01, D-glucose decreased significantly at heat-stress temperatures. CC7-SSA01 anemones showed the highest abundance for most metabolites at ambient temperature and normal salinity (36). At heat-stress temperatures, this pattern shifted and the highest levels for the majority of metabolites were at intermediate salinity (Fig. 3A). Notably, floridoside was not measured (below detection levels) in CC7-SSA01 (Fig. 3C), and we did not find any other osmolyte that showed increased abundance with increasing salinity. As such, only floridoside in H2-SSB01 featured increased levels over increased salinities, consistent with the increase in thermotolerance observed for this host–endosymbiont pairing. Of note, floridoside levels increased with salinity for algal endosymbiont-normalized as well as total protein-normalized concentrations (Fig. 3B; Fig. S4). As such, differences in floridoside levels were not the result of changes in endosymbiont density. Conversely, CC7-SSA01, which is not amenable to a salinity-conveyed thermotolerance, also did not harbor any measured osmolytes that increased in abundance at high salinity.

## DISCUSSION

Prompted by previously published work that high salinity conveys thermotolerance in Aiptasia (Gegner et al., 2017) and that the osmolyte floridoside is highly abundant in coral holobionts at high salinity (Ochsenkühn et al., 2017), we here assessed the presence of a mechanistic link between osmoadaptation and thermotolerance in the cnidarian-algal endosymbiosis. Using different host–endosymbiont pairings of Aiptasia anemones, we could show that for the pairing H2-SSB01, increased thermotolerance and reduced bleaching under heat stress at high salinity resulted in reduced ROS leakage concomitant with increased floridoside levels. This lends further support to the proposed dual function of floridoside as an osmolyte and ROS scavenger in algal endosymbionts (Ochsenkühn et al., 2017), due to its antioxidative capabilities (Li et al., 2010; Pade et al., 2015). In particular, since floridoside is the only measured carbohydrate osmolyte that increased abundance levels with increasing salinity, it is a likely candidate (among other metabolites not measured here, e.g. glutathione) to reduce ROS levels, thereby conveying thermotolerance at high salinity. Nevertheless, a direct functional link remains to be established, e.g. via knockdown or overexpression of the underlying genes. Since corals as well as Aiptasia are lacking the genes to synthesize floridoside (Pade et al., 2015; Ochsenkühn et al., 2017), such functional testing would need to be carried out in the algal endosymbiont, which seems particularly intractable to genetic manipulation (Chen et al., 2019). Besides such limitations, our data suggest a link between osmoadaptation and thermotolerance in the cnidarian-algal symbiosis with implications for the importance of osmoadaptation to stress resilience.

### The osmolyte floridoside links osmoadaptation with thermotolerance as a putative broadly present mechanism

Our results reveal a differential response of the two Aiptasia strains to heat stress under different salinities. Symbiotic H2-SSB01 anemones exhibited higher symbiont loss and reduction in photosynthetic efficiency during heat stress than symbiotic CC7-SSA01 Aiptasia. Further, reduced abundance of most of the carbohydrates points towards increased metabolism and energy consumption during heat stress in H2-SSB01. By comparison, carbohydrate levels in CC7-SSA01 were more stable under heat stress. Interestingly, however, H2-SSB01 became more thermotolerant with increasing salinities, whereas CC7-SSA01 did not seem to respond to an increase in salinity. This makes these Aiptasia strains a good model system to study differences in osmoadaptation-related thermotolerance.

At this point, the underlying cause of the difference in salinity-conveyed thermotolerance remains unclear. In a recent study by Cziesielski et al. (2018), the authors showed that different Aiptasia strains harbor similar antioxidant capacities, but that observed differences of ROS levels *in hospite* were endosymbiont-driven. Indeed, bleaching susceptibility in our experiments directly aligned with changes in ROS leakage of the endosymbionts during heat stress. Whereas CC7-SSA01 showed overall low ROS leakage across salinities, supporting a high inherent thermotolerance, H2-SSB01 showed reduced ROS leakage at increasing salinities. Thus, endosymbiont identity seems to, at least partially, determine thermotolerance of holobionts. Further, differences in symbiotic ROS leakage are likely not only underlying the differential bleaching susceptibility between host–endosymbiont pairings, but also play a role in the salinity-conveyed thermotolerance. In the case of H2-SSB01, the decrease of ROS leakage at increasing salinities during heat stress aligns with increased levels of the osmolyte floridoside, which was previously suggested to be an osmolyte of coral algal endosymbionts (Ochsenkühn et al., 2017). Ochsenkühn et al. (2017) further hypothesized that floridoside might play an important role in countering ROS arising from salinity and heat stress, given that it is a potent antioxidant in many marine algae (Li et al., 2010; Martinez-Garcia and van der Maarel, 2016). Our results corroborate this notion and mechanistically link osmoadaptation with thermotolerance via increased floridoside levels at high salinities, whereby floridoside plays a dual role as an osmolyte and ROS scavenger. Importantly, floridoside was only measured in H2-SSB01 and showed increased levels at increased salinities, in line with reduced ROS leakage of the algal endosymbionts. By contrast, floridoside was not detectable in CC7-SSA01, which neither exhibited reduced ROS leakage nor increased thermotolerance at high salinities.

Future experiments could test for the presence of salinity-conveyed thermotolerance, reduced ROS leakage of the algal endosymbionts, and floridoside abundance levels in reversed host–endosymbiont pairings, i.e. H2-SSA01 and CC7-SSB01. This would also clarify the relative contribution of host and endosymbiont, respectively the importance of host and endosymbiont identity. While the Aiptasia system explicitly allows to test the same host with different endosymbionts and vice versa (Voolstra, 2013), one must acknowledge that the performance of native host endosymbiont associations are optimized and often exceed non-native host endosymbiont associations (Matthews et al., 2017; Rädecker et al., 2018). Therefore, results from such experiments may still be ambiguous. At this point, data obtained from Red Sea corals in a pilot study (data not shown) support the idea that salinity-conveyed thermotolerance might be a wider phenomenon, hinting towards a broadly present mechanism. Indeed, studies from plants grown at high salinities have shown an increased temperature tolerance and this was attributed to an increased production of osmolytes (Lu et al., 2003; Rivero et al., 2014). It is important to note that floridoside is only one of many molecules that may link osmoadaptation with thermotolerance in symbiotic cnidarians. As such, we rather argue for the importance of the mechanistic link between osmoadaptation and thermotolerance, than for any particular osmolyte. There is a number of osmolytes that may contribute to the salinity-conveyed thermotolerance besides floridoside, such as dimethylsulphoniopropionate (DMSP) or amino acids (Mayfield and Gates, 2007; Yancey et al., 2010; Ochsenkühn et al., 2017).

### A new perspective for corals in extreme environments

The extraordinary thermotolerance of corals from the Red Sea and Persian/Arabian Gulf has been demonstrated in a number of studies (Fine et al., 2013; Hume et al., 2016; Krueger et al., 2017; Osman et al., 2018). Importantly, D'Angelo et al. (2015) showed that superior heat tolerance is lost when corals from the Persian/Arabian Gulf are exposed to reduced salinity levels. While this may argue for strong local adaptation to high temperature and the exceptionally high salinity in the Persian/Arabian Gulf, it may, at least partially, relate to the here demonstrated link between salinity and thermotolerance. This is further supported by the higher heat tolerance of corals in the northern Red Sea (Osman et al., 2018) and the Gulf of Aqaba (Fine et al., 2013), in comparison to their central and southern Red Sea counterparts, in line with the northern Red Sea harboring much higher salinity levels (≥41) than the central and southern counterparts (36) (Ngugi et al., 2012). As such, it remains to be determined whether salinity levels may affect the stress resilience of corals on a global scale. Notably, our results highlight the complexity of interactions underlying holobiont resilience. Besides algal endosymbionts, other microbiome members such as bacteria and archaea should also be taken into account, as they may rapidly respond to salinity (Röthig et al., 2016) and may contribute to the thermotolerance of the coral holobiont (Ziegler et al., 2017).

### Conclusion

Recent work showing reduced bleaching at high salinities and high levels of floridoside, an osmolyte with antioxidative capabilities, at high salinities, encouraged us to assess a link between osmoadaptation and thermotolerance in symbiotic cnidarians. Exposing the coral model Aiptasia to heat at different salinities confirms increased thermotolerance and reduced bleaching at high salinity, manifested by reduced ROS leakage. The decrease of ROS leakage followed increased levels of the ROS-scavenging osmolyte floridoside under increasing salinities, thus, arguing for a mechanistic link between osmoadaptation and thermotolerance in the cnidarian-dinoflagellate endosymbiosis. Our results may help to explain the extraordinarily high thermotolerance of corals from the Arabian Seas and may hold implications about the response of corals to rising sea surface temperatures considering salinity as a contributing factor. Future studies should assess whether salinity-conveyed thermotolerance is a mechanism that is present in corals and whether other osmolytes (and which ones) may be important and play a role in salinity-conveyed thermotolerance.

## MATERIALS AND METHODS

### Aiptasia experimental procedures

#### Anemone rearing, experimental setup, and sample processing

We used symbiotic and aposymbiotic anemones of the clonal Aiptasia strains H2 and CC7 as previously described (Gegner et al., 2017). H2 anemones were associated with their native endosymbionts type B1 [strain SSB01, species *Breviolum minutum* (Xiang et al., 2013; Baumgarten et al., 2015)], referred to as H2-SSB01, and CC7 anemones were associated with their native endosymbionts type A4 [strain SSA01, species *Symbiodinium linucheae* (Bieri et al., 2016)], referred to as CC7-SSA01. A subset of anemones from these host–endosymbiont pairings were rendered aposymbiotic following the menthol bleaching method by Matthews et al. (2016) and treated as their symbiotic counterparts. All anemones were kept at a 12 h light:12 h dark cycle at 30–40 μmol m^−2^ s^−1^.

For the experiment, anemones were kept at ambient temperature (25°C) at three salinities: low (36), intermediate (39), and high (42) for 10 days to acclimate (Fig. 1A). A total of 252 anemones were used (symbiotic: 18 animals×2 host–endosymbiont pairings×3 salinities×2 temperatures=216; aposymbiotic: 3 animals×2 hosts×3 salinities×2 temperatures=36). Symbiotic and aposymbiotic animals were transferred to individual wells of six-well plates (water volume of 7 ml) and wrapped in a see-through plastic bag containing wet wipes to minimize evaporation. Salinities were monitored throughout the entire experiment using a refractometer (Aqua Medic GmbH, Germany). Wells were cleaned every second day with a cotton-swap and the water was exchanged. Feeding was ceased with the beginning of the acclimation. The experimental salinities were achieved by using autoclaved Red Sea seawater diluted with ddH_2_O to the lowest salinity (36) and subsequently adjusted with NaCl to the desired salinities.

After the acclimation phase half of the symbiotic and aposymbiotic anemones were subjected to heat stress and the remaining half were kept at ambient temperatures. For the heat stress, anemones were moved to another incubator with identical settings where the temperature was ramped from 25°C to 34°C over the course of 10 h (1°C h^−1^ increment) and was held for the remainder of the experiment. The experiment was terminated after 6 days when H2-SSB01 anemones in the lowest salinity (36) appeared completely bleached visually, i.e. translucent. At the same time, anemones from the control were collected. Individual Aiptasia were rinsed twice with ddH20, briefly drained of excess water, transferred into single cryotubes and immediately snap frozen in liquid nitrogen and stored at −80°C until further processing.

### Photosynthetic efficiency over the course of the heat stress

Light-adapted photosynthetic efficiency (ΔF/Fm′) of photosystem II (PSII) for each symbiotic anemone (*N*=12 per experimental condition and host–endosymbiont pairing, total of *N*=144) was measured daily (5 h into the light phase) for the period of the experiment using a diving Pulse Amplitude Modulated (PAM) fluorometer (Walz, Germany) (see Fig. S1B).

### Algal endosymbiont counts

Snap frozen anemones (see above) were thawed on ice and homogenized as previously described (Krediet et al., 2015; Gegner et al., 2017). Algal endosymbiont cell counts with three technical replicates per sample were obtained using a BD LSR Fortessa cell analyzer (BD Biosciences, USA). Algal endosymbionts were discriminated from anemone host cells and debris using a combination of forward/side scatter as well as chlorophyll fluorescence. Counts were then normalized to the host protein of each anemone using the PierceBCAassay (Thermo Fisher Scientific) according to the manufacturer's instructions.

### ROS leakage measurement from algal endosymbionts

The used protocol was adapted from Cziesielski et al. (2018): shorter incubation times were used and antibiotic treatments were omitted. A 2-day sampling time point was chosen to obtain ROS measurements at the peak of heat stress, but before the anemones started to visually bleach. This is because measuring ROS release after the expulsion of the majority of endosymbionts may offer a poor reflection of the underlying causes of bleaching. Concomitant with the peak in heat stress, we measured a decreased photosynthetic efficiency at the 2-day time point, but no visible bleaching.

Symbiotic Aiptasia, i.e. H2-SSB01 and CC7-SSA01 (*N*=6 per experimental condition and host–endosymbiont pairing) were subjected to the same experimental conditions as described above. After 2 days at ambient and heat stress temperatures anemones were homogenized in seawater that was adjusted to their respective salinity (i.e., 36, 39, and 42). The algal endosymbiont fraction was isolated by centrifugation for 5 min at 3000× ***g*** and washed twice with 1× PBS. The endosymbiont pellet was then re-suspended in seawater that was adjusted to the respective salinity (36, 39, 42) and further incubated for 2 h at the respective temperature (25°C or 34°C). Next, CellROX Orange Reagent (Thermo Fisher Scientific) was added to a final concentration of 5 µM, vortexed, then centrifuged. Supernatant was finally transferred to a black 96-well plate, which was incubated for 30 min at 37°C in the dark following the manufacturer's protocol. Fluorescence was measured in a spectrophotometer (SpectraMax Paradigm, Molecular Devices LLC, USA) at 545/565 nm to quantify the amount of leaked ROS to the medium. Recorded fluorescence intensity was normalized to the number of endosymbiont cells, determined using a BD LSR Fortessa cell analyzer (Table S11).

#### Analysis and characterization of the carbohydrate and fatty acid fraction using targeted GC-MS

The step-by-step protocol for the sample preparation, metabolite extraction, and derivatization based on Ochsenkühn et al. (2017) used here is available online at protocols.io (Gegner et al., 2018). Briefly, snap-frozen Aiptasia anemones were resuspended in 1 ml ddH_2_O and disrupted by tip sonication at 7 W for 2 min on ice in a cold room. Homogenates were then centrifuged at 4000× ***g*** for 20 min at 4°C to remove cell debris. After that, nine parts of −20°C ethanol were added to one part supernatant to precipitate all proteins, DNA, and RNA. The mixture was then centrifuged at 20,000× ***g*** for 20 min at 4°C. The precipitated pellet was saved for total protein quantification and later analyzed using the PierceBCAassay (Thermo Fisher Scientific) according to manufacturer's instructions. The supernatant was transferred to a new falcon tube, stored at −80°C overnight, and subsequently lyophilized using a FreezeDryer (Ultradry, USA). The dried samples were then dissolved in 500 µl ddH_2_O, spiked with 10 µl internal standard [HBA in ddH_2_O (1 µg µl^−1^)] transferred into GC vials, and dried again using a concentrator system (Labconco Centrivap Complete, USA). For derivatization of the samples, 50 μl of MOX reagent (2% methoxamine HCL in pyridine) was added and the solution heated to 75°C for 1 h. After that, 100 µl of MSTFA [N-methyl-N-(trimethylsilyl)trifluoroacetamide, 1% trimethylchlorosilane; Thermo Fisher Scientific] was added to each vial, heated again to 75°C for 1 h and filtered. The extracted metabolites were then analyzed by a GC-MS system [GC (Agilent 7890A) and MS (Agilent 5975C)], and quantified using a set of determined standard curves for glucose (99.5%; Sigma-Aldrich), sucrose (99.5%; Sigma-Aldrich), glycerol (≥99.5%, ACS Reagent–grade; Sigma-Aldrich) and glycine (ACS reagent, ≥98.5%; Sigma-Aldrich). All GC-MS data were processed (background subtraction, peak picking, and integration) using OpenChrom 1.1.0 (Diels) and identified using MS ionization spectra (NISTMS Software 2.0, Agilent Technologies, USA). Metabolite levels were corrected to the GC-MS internal standard hydroxy benzylic acid (HBA), followed by conversion to pmol using molar masses. Metabolite levels were normalized to the total protein content of the holobiont (Table S12). In addition, floridoside levels were normalized to obtained endosymbiont densities (Fig. 3B; Table S13), given that they are the producers of this osmolyte in the cnidarian-algal endosymbiosis (Pade et al., 2015; Ochsenkühn et al., 2017) and that floridoside was only detected in symbiotic holobionts. Of note, algal endosymbiont densities from the tissue slurry used for metabolite characterization were not recorded. Thus, metabolite levels based on total protein normalization were converted into metabolite levels per algal endosymbiont cell using endosymbiont densities obtained from the set of Aiptasia used to produce Fig. S1 (Table S9) assuming that total protein content largely resembles host protein content.

#### Statistical analyses

The effect of ambient and heat stress temperatures (25°C and 34°C) at low (36), intermediate (39), and high (42) salinities on endosymbiont densities and ROS leakage was tested using two-way analysis of variance (ANOVA) followed by pairwise Tukey post-hoc tests using JMP Pro 13.1.0 (SAS Campus Drive, USA).

To analyze differences between PAM measurements at ambient and heat stress temperatures (25°C and 34°C) and across salinities (36, 39, 42), the non-parametric Steel-Dwass test was used using JMP Pro 13.1.0 (SAS Campus Drive, USA).

To analyze differences between metabolite levels determined via a targeted GC-MS approach, we used PRIMER-E (Clarke and Gorley, 2015) to test for differences of mean-centered metabolite compositions between host–endosymbiont pairings using a PERMANOVA with the factors host (H2 and CC7) and symbiotic state (symbiotic and aposymbiotic) at the two temperatures (25°C and 34°C) at low (36), intermediate (39), and high (42) salinities. Subsequent analyses of each Aiptasia host–endosymbiont pairing were conducted using MetaboAnalyst 4.0 (Chong et al., 2018). To test for significant metabolite level differences across salinities and temperatures, we used two-way ANOVAs with false discovery rate (FDR)-adjusted *P-*values (*P*<0.05) using the factors temperature (25°C and 34°C) and salinity (36, 39, 42) for each host pairing. To subsequently determine the specific salinity at which floridoside levels normalized to total protein content in H2-SSB01 were significantly different for each temperature (25°C and 34°C), we applied non-parametric Kruskal–Wallis (*P*<0.05) using JMP Pro 13.1.0 (SAS Campus Drive, USA). We followed the same procedure for the floridoside levels normalized per endosymbiont cell.

## Supplementary Material

Supplementary information
