# Supplementary material for: High levels of floridoside at high salinity link osmoadaptation with bleaching susceptibility in the cnidarian-algal endosymbiosis
Source: Biol Open. 2019 Dec 16;8(12):bio045591. doi: 10.1242/bio.045591 (PMC6918757; doi:10.1242/bio.045591)
Supplement: Supplementary information [file biolopen-8-045591-s1.pdf]

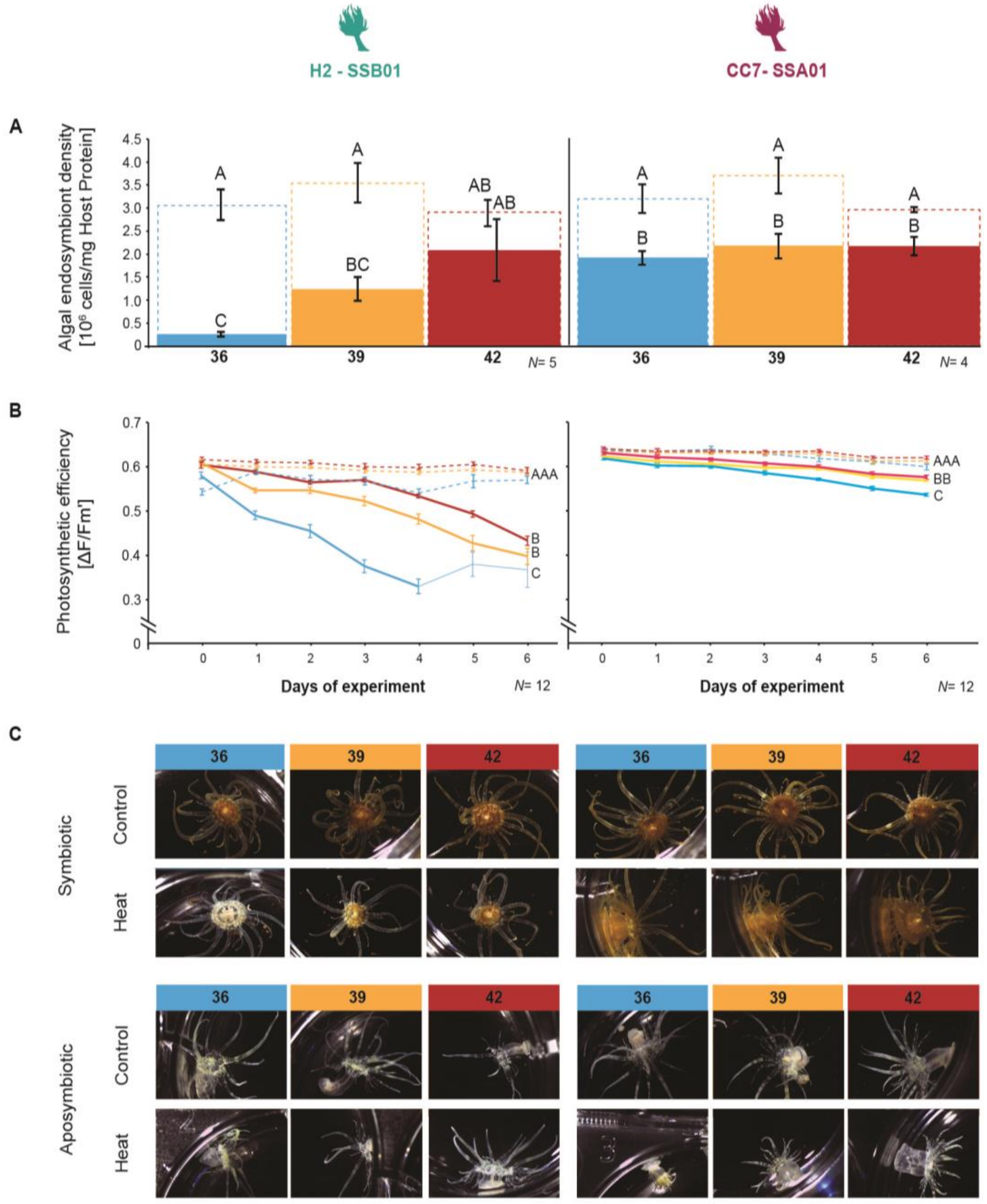

Figure S1. Effect of different salinities on heat stress-induced bleaching of H2 and CC7 Aiptasia anemones. (A) Algal endosymbiont densities of host-endosymbiont pairings at ambient (dashed bars, data from Gegner et al. (2017)) and heat stress (solid bars) temperatures at low (36), intermediate (39), and high (42) salinities. Different letters above bars indicate significant differences between groups (Tukey post-hoc, $p < 0.05$). Error bars show the standard error of the mean (SE). **(B)** Light-adapted photosynthetic efficiency over the period of the experiment. Dashed lines depict animals at ambient

(control) temperature (25 °C); solid lines depict animals under heat stress (34 °C). The faded blue line indicates the days for which a robust measurement could only be retrieved from 2 of 12 animals. Different letters indicate significant differences between salinity and temperature treatments after 6 days of experiment (non-parametric, Steel-Dwass, $p < 0.05$). Error bars show the standard error of the mean (SE). **(C)** Pictures of representative symbiotic and aposymbiotic anemones at ambient (Control) and heat stress temperature (Heat) across different salinities after 6 days of the experiment.

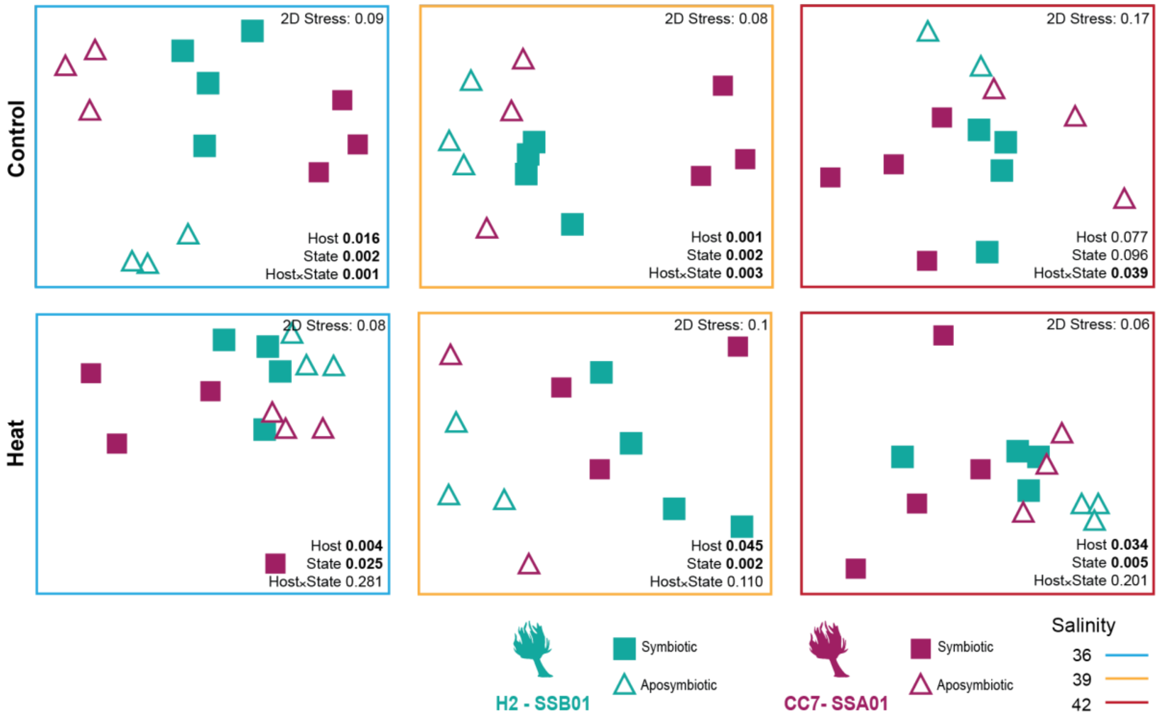

Figure S2. Multidimensional scaling (MDS) plots of metabolite profiles of H2 and CC7 Aiptasia anemones at ambient (Control) and heat stress (Heat) temperatures at low (36), intermediate (39), and high (42) salinities. PERMANOVA was used to test for significant difference between anemone hosts (H2 and CC7), symbiotic states (H2-SSB01 vs. H2-aposymbiotic and CC7-SSA01 vs. CC7-aposymbiotic), and interactions thereof (Host x State); p-values are provided in bottom-right of each panel.

Biology Open • Supplementary information

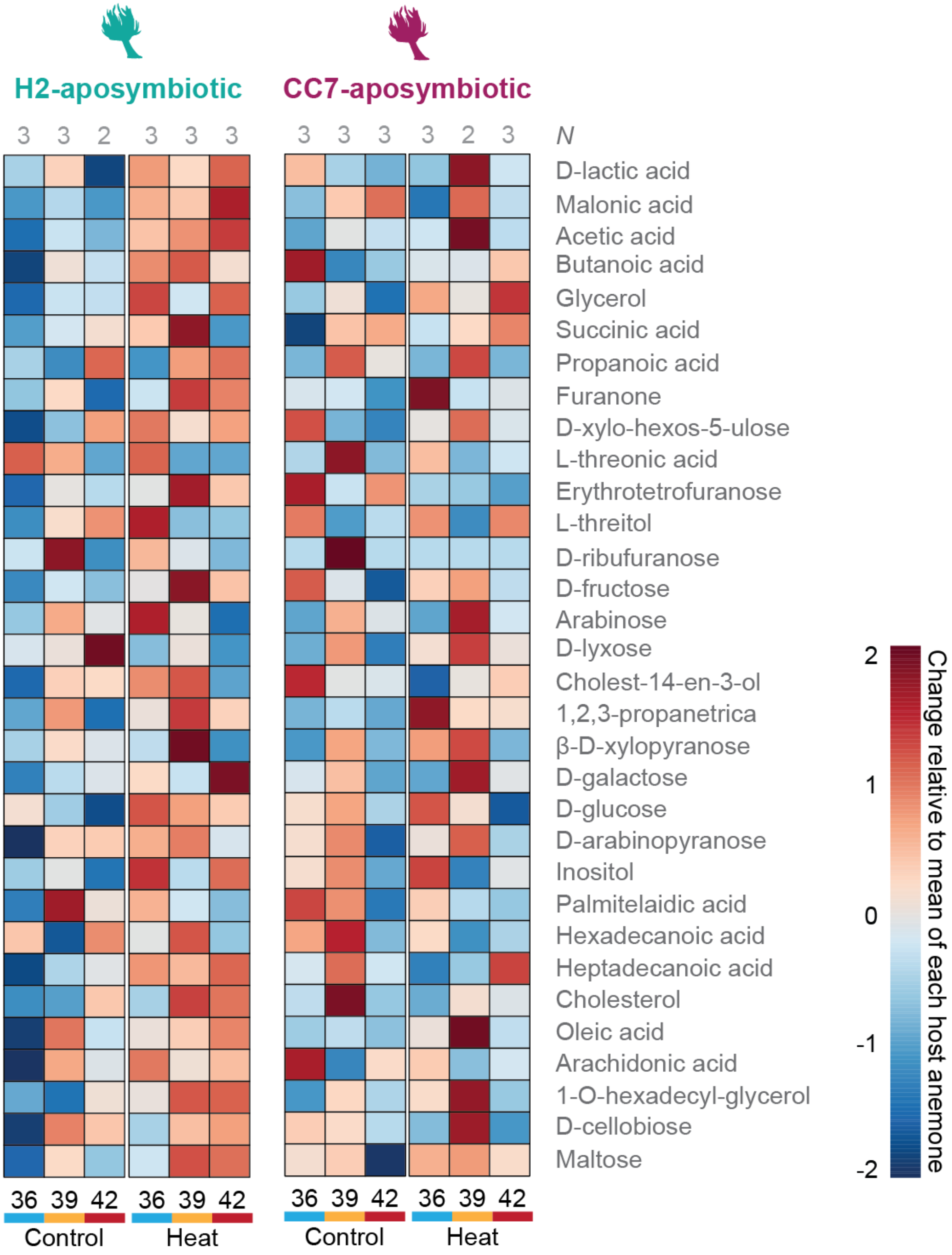

Figure S3. Metabolite levels of aposymbiotic H2 and CC7 Aiptasia anemones normalized to total protein content. Heatmaps show metabolite levels at ambient (Control) and heat stress (Heat) temperatures at low (36), intermediate (39), and high (42) salinity. Levels of metabolites were not significantly different across salinities and temperatures for each host anemone (2-Way ANOVAs, all $p > 0.05$).

Biology Open • Supplementary information

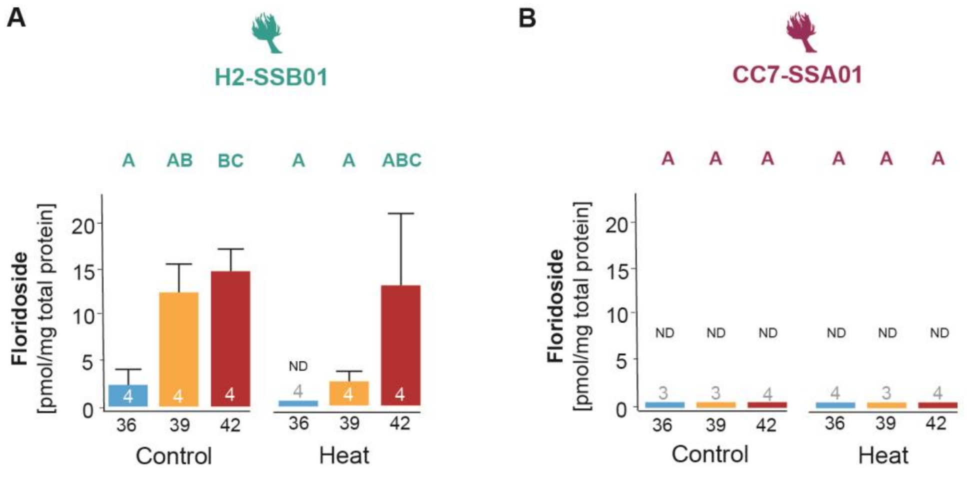

Figure S4. Floridoside levels of H2-SSB01 and CC7-SSA01 at ambient (Control) and heat stress (Heat) temperatures at low (36), intermediate (39), and high (42) salinities normalized to total protein. Different letters above bars indicate significant differences between groups (Kruskal-Wallis, $p < 0.05$). Number of replicates is indicated above/within bars. ND = not detected. Error bars show the standard error of the mean (SE).

Table S1. Statistical test results on retained algal endosymbionts normalized to host protein (1-Way ANOVA). The purpose was to test for a significant effect of low (36), intermediate (39), and high (42) salinities in (A) H2-SSB01 and (B) CC7-SSA01. To elucidate significant differences, a pairwise Tukey post-hoc test was conducted for H2-SSB01. Bold values indicate $p < 0.05$.

(A) H2-SSB01

Source of variation	DF	Sum of squares	F	p-Value
Between groups	2	10308.850	5.188	**0.024**
Within groups	12	11922.239		
Total	14	22231.090		

Tukey post-hoc

Comparison		Score Mean Difference	p-Value
Sal39	Sal36	26.482	0.407
Sal42	Sal36	63.904	**0.019**
Sal42	Sal39	37.421	0.188

(B) CC7-SSA01

Source of variation	DF	Sum of squares	F	p-Value
Between groups	2	539.124	2.708	0.120
Within groups	9	895.740		
Total	11	1434.864		

Biology Open • Supplementary information

Table S2. Statistical test results on Aiptasia algal endosymbiont density (2-Way ANOVA). The purpose was to test for a significant difference between ambient (25 °C) and heat stress (34 °C) temperatures at low (36), intermediate (39), and high (42) salinities in (A) H2-SSB01 and (B) CC7-SSA01. To elucidate significant differences, a pairwise Tukey post-hoc test was conducted for H2-SSB01. Bold values indicate $p < 0.05$.

(A) H2-SSB01

Source of variation	DF	Sum of squares	F	p-Value
Salinity (Sal)	2	4.011e+12	2.689	0.088
Temperature (Temp)	1	2.920e+13	39.139	**<0.001**
Sal x Temp	2	5469e+12	3.665	**0.041**

Tukey post-hoc

Comparison		Score Mean Difference	p-Value
Sal39, Temp25	Sal36, Temp34	3283344	**<0.001**
Sal36, Temp25	Sal36, Temp34	2810596	**<0.001**
Sal42, Temp25	Sal36, Temp34	2624302	**<0.001**
Sal39, Temp25	Sal39, Temp34	2307722	**0.004**
Sal36, Temp25	Sal39, Temp34	1834974	**0.028**
Sal42, Temp34	Sal36, Temp34	1823430	**0.029**
Sal42, Temp25	Sal39, Temp34	1648680	0.059
Sal39, Temp25	Sal42, Temp34	1459914	0.118
Sal36, Temp25	Sal42, Temp34	987166	0.480
Sal39, Temp34	Sal36, Temp34	975622	0.493
Sal42, Temp34	Sal39, Temp34	847808	0.636
Sal42, Temp25	Sal42, Temp34	800872	0.688
Sal39, Temp25	Sal42, Temp25	659042	0.829
Sal39, Temp25	Sal36, Temp25	472748	0.951
Sal36, Temp25	Sal42, Temp25	186294	0.999

(B) CC7-SSA01

Source of variation	DF	Sum of squares	F	p-Value
Salinity (Sal)	2	7.876e+11	1.467	0.257
Temperature (Temp)	1	9.322e+12	34.719	**<0.001**
Sal x Temp	2	6.535e+11	1.217	0.319

Table S3. Statistical test results on light-adapted photosynthetic efficiency (∆Fv/Fm') of symbiotic Aiptasia (non-parametric Steel-Dwass). The purpose was to test for a significant difference between ambient (25 °C) and heat stress (34 °C) temperatures at low (36), intermediate (39), and high (42) salinities on the last day of the experiment in (A) H2-SSB01 and (B) CC7-SSA01. Bold values indicate $p < 0.05$.

(A) H2-SSB01

Comparison		Score Mean Difference	p-Value
Temp34, Sal42	Temp34, Sal36	10.583	**0.002**
Temp34, Sal39	Temp34, Sal36	9.417	**0.011**
Temp25, Sal42	Temp25, Sal36	4.917	0.530
Temp34, Sal42	Temp34, Sal39	3.667	0.801
Temp25, Sal39	Temp25, Sal36	2.917	0.915
Temp25, Sal42	Temp25, Sal39	1.750	0.991
Temp34, Sal39	Temp25, Sal36	-11.750	**<0.001**
Temp34, Sal36	Temp25, Sal36	-11.917	**<0.001**
Temp34, Sal36	Temp25, Sal39	-11.917	**<0.001**
Temp34, Sal36	Temp25, Sal42	-11.917	**<0.001**
Temp34, Sal39	Temp25, Sal39	-11.917	**<0.001**
Temp34, Sal39	Temp25, Sal42	-11.917	**<0.001**
Temp34, Sal42	Temp25, Sal36	-11.917	**<0.001**
Temp34, Sal42	Temp25, Sal39	-11.917	**<0.001**
Temp34, Sal42	Temp25, Sal42	-11.917	**<0.001**

(B) CC7-SSA01

Comparison		Score Mean Difference	p-Value
Temp34, Sal39	Temp34, Sal36	11.917	**<0.001**
Temp34, Sal42	Temp34, Sal36	11.917	**<0.001**
Temp25, Sal42	Temp25, Sal36	6.667	0.189
Temp25, Sal39	Temp25, Sal36	4.333	0.663
Temp34, Sal42	Temp34, Sal39	3.500	0.831
Temp25, Sal42	Temp25, Sal39	2.083	0.979
Temp34, Sal42	Temp25, Sal39	-6.417	0.225
Temp34, Sal39	Temp25, Sal36	-8.417	**0.041**
Temp34, Sal42	Temp25, Sal39	-10.000	**0.007**
Temp34, Sal39	Temp25, Sal39	-10.583	**0.003**
Temp34, Sal42	Temp25, Sal42	-11.000	**0.002**
Temp34, Sal36	Temp25, Sal36	-11.667	**<0.001**
Temp34, Sal36	Temp25, Sal39	-11.917	**<0.001**
Temp34, Sal36	Temp25, Sal42	-11.917	**<0.001**
Temp34, Sal39	Temp25, Sal42	-11.917	**<0.001**

Biology Open • Supplementary information

Table S4. Statistical test results on the relative change of ROS leakage per algal endosymbiont cell isolated from symbiotic Aiptasia (1-Way ANOVA). The purpose was to test for a significant difference across low (36), intermediate (39), and high (42) salinities for (A) SSB01 and (B) SSA01. To elucidate significant differences, a pairwise Tukey post-hoc test was conducted for SSB01. Bold values indicate $p < 0.05$.

(A) SSB01

Source of variation	DF	Sum of squares	F	p-Value
Between groups	2	6.607	12.056	**<0.001**
Within groups	14	3.836		
Total	16	10.443		

Tukey post-hoc

Comparison		Score Mean Difference	p-Value
Sal39	Sal36	-0.745	0.081
Sal42	Sal36	-1.552	**<0.001**
Sal42	Sal39	-0.807	**0.045**

(B) SSA01

Source of variation	DF	Sum of squares	F	p-Value
Between groups	2	0.025	0.801	0.468
Within groups	14	0.218		
Total	16	0.243		

Table S5. Statistical test results on the composition of Aiptasia metabolite profiles (PERMANOVA) normalized to total protein content. The purpose was to test for a significant difference between low (36), intermediate (39), and high (42) salinities at (A) ambient (25 °C) and (B) heat stress (34 °C) temperatures considering the factors host (H2 and CC7) and symbiotic state (symbiotic, aposymbiotic). Bray–Curtis dissimilarity and 999 permutations were used for this analysis. Bold values indicate p < 0.05.

(A) Ambient temperature (25°C)

Salinity 36	DF	Sum of squares	Pseudo-F	p-Value
Host	1	814.040	2.652	**0.016**
State	1	2558.700	8.337	**0.002**
Host x State	1	2099.500	6.801	**0.001**

Salinity 39	DF	Sum of squares	Pseudo-F	p-Value
Host	1	2676.900	7.589	**0.001**
State	1	3728.300	10.570	**0.002**
Host x State	1	1990.900	5.644	**0.003**

Salinity 42	DF	Sum of squares	Pseudo-F	p-Value
Host	1	1589.900	1.773	0.077
State	1	1504.200	1.678	0.096
Host x State	1	1926.600	2.149	**0.039**

(B) Heat stress (34°C)

Salinity 36	DF	Sum of squares	Pseudo-F	p-Value
Host	1	1649.500	3.137	**0.004**
State	1	1775.500	3.377	**0.025**
Host x State	1	669.120	1.273	0.281

Salinity 39	DF	Sum of squares	Pseudo-F	p-Value
Host	1	1158.000	2.131	**0.045**
State	1	3078.800	5.666	**0.002**
Host x State	1	988.550	1.819	0.110

Salinity 42	DF	Sum of squares	Pseudo-F	p-Value
Host	1	1903.600	2.460	**0.034**
State	1	3997.500	5.165	**0.005**
Host x State	1	1044.900	1.350	0.201

Biology Open • Supplementary information

Table S6. Statistical test results on Aiptasia metabolite levels (2-Way ANOVA) normalized to total protein content. The purpose was to test for a significant difference between ambient (25 °C) and heat stress (34 °C) temperatures at low (36), intermediate (39), and high (42) salinities in (A) H2-SSB01 and (B) CC7-SSA01. Reported are only significant metabolites. Bold values indicate FDR-corrected p < 0.05.

(A) H2-SSB01

Metabolite	Temperature		Salinity		Interaction	
	F	p-Value	F	p-Value	F	p-Value
D-glucose	**41.821**	**<0.001**	2.754	0.171	4.913	0.147
L-threitol	**13.876**	**0.026**	**6.259**	**0.042**	5.938	0.147
Oleic acid	7.245	0.085	**7.539**	**0.042**	4.117	0.151
Palmitelaidic acid	2.402	0.369	**6.364**	**0.042**	5.518	0.147
Floridoside	2.235	0.370	**7.111**	**0.042**	0.917	0.507
1-O-hexadecylglycerol	1.599	0.472	**10.408**	**0.034**	4.777	0.147
Furanone	0.017	0.923	**8.384**	**0.042**	3.209	0.164
Hexadecanoic acid	0.010	0.923	**6.437**	**0.042**	3.19	0.164

(B) CC7-SSA01

Metabolite	Temperature		Salinity		Interaction	
	F	p-Value	F	p-Value	F	p-Value
D-glucose	**16.855**	**0.030**	1.330	0.837	4.572	0.180
Arachidonic acid	8.383	0.122	5.047	0.337	**13.057**	**0.008**
L-threonic acid	4.323	0.270	6.204	0.337	**14.364**	**0.008**

Biology Open • Supplementary information

Biology Open (2019): doi:10.1242/bio.045591: Supplementary information

Table S7. Statistical test results on floridoside levels for H2 Aiptasia anemones (Kruskal-Wallis) normalized to total protein content. The purpose was to test for a significant difference between low (36), intermediate (39), and high (42) salinities as well as ambient (25 °C) and heat stress (34 °C) temperatures. Bold values indicate $p < 0.05$.

Comparison		Score Mean Difference	p-Value
Sal39, Temp25	Sal36, Temp34	3.750	**0.021**
Sal36, Temp25	Sal36, Temp34	-1.750	0.186
Sal42, Temp25	Sal36, Temp34	3.750	**0.021**
Sal39, Temp25	Sal39, Temp34	-3.750	**0.030**
Sal36, Temp25	Sal39, Temp34	1.250	0.468
Sal42, Temp34	Sal36, Temp34	3.750	**0.021**
Sal42, Temp25	Sal39, Temp34	3.750	**0.030**
Sal39, Temp25	Sal42, Temp34	-0.250	0.885
Sal36, Temp25	Sal42, Temp34	2.750	0.110
Sal39, Temp34	Sal36, Temp34	3.750	**0.021**
Sal42, Temp34	Sal39, Temp34	3.250	0.061
Sal42, Temp25	Sal42, Temp34	-1.250	0.471
Sal39, Temp25	Sal42, Temp25	1.250	0.471
Sal39, Temp25	Sal36, Temp25	3.250	0.059
Sal36, Temp25	Sal42, Temp25	3.750	**0.029**

Biology Open • Supplementary information

Table S8. Statistical test results on floridoside levels for H2 Aiptasia anemones (Kruskal-Wallis) normalized to algal endosymbiont densities. The purpose was to test for a significant difference between low (36), intermediate (39), and high (42) salinities as well as ambient (25 °C) and heat stress (34 °C) temperatures. Bold values indicate $p < 0.05$.

Comparison		Score Mean Difference	p-Value
Sal39, Temp25	Sal36, Temp34	3.750	**0.021**
Sal36, Temp25	Sal36, Temp34	-1.750	0.186
Sal42, Temp25	Sal36, Temp34	3.750	**0.021**
Sal39, Temp25	Sal39, Temp34	-1.750	0.312
Sal36, Temp25	Sal39, Temp34	2.250	0.191
Sal42, Temp34	Sal36, Temp34	3.750	**0.021**
Sal42, Temp25	Sal39, Temp34	3.250	0.061
Sal39, Temp25	Sal42, Temp34	0.250	0.885
Sal36, Temp25	Sal42, Temp34	3.250	0.059
Sal39, Temp34	Sal36, Temp34	3.750	**0.021**
Sal42, Temp34	Sal39, Temp34	1.750	0.312
Sal42, Temp25	Sal42, Temp34	-0.750	0.665
Sal39, Temp25	Sal42, Temp25	1.750	0.312
Sal39, Temp25	Sal36, Temp25	2.750	0.110
Sal36, Temp25	Sal42, Temp25	3.750	**0.029**

Biology Open • Supplementary information

Table S9. Algal endosymbiont cell counts determined by flow cytometry and normalized to host protein for Aiptasia host-endosymbiont pairings H2-SSB01 and CC7-SSA01. Each row depicts a replicate anemone.

Click here to Download Table S9

Table S10. Photosynthetic efficiency measurements for Aiptasia host-endosymbiont pairings H2-SSB01 and CC7-SSA01. Each row depicts a replicate anemone.

Click here to Download Table S10

Table S11. **ROS leakage (CellROX fluorescence) measurements for algal endosymbiont fraction from Aiptasia host-endosymbiont pairings H2-SSB01 and CC7-SSA01.** Each row depicts a replicate anemone.

Click here to Download Table S11

Biology Open • Supplementary information

Table S12. Targeted GC-MS metabolite levels of Aiptasia host-endosymbiont pairings H2-SSB01 and CC7-SSA01 in their symbiotic and aposymbiotic state normalized to total protein content. Metabolite levels were normalized to the GC-MS internal standard hydroxy benzylic acid (HBA), then to mg of total protein, followed by conversion to pmol using molar masses, which yielded metabolite levels in pmol mg^{-1} total protein. Each row depicts a replicate anemone.

Click here to Download Table S12

Table S13. Relative floridoside levels converted from total protein-normalized floridoside levels using algal endosymbiont densities obtained from the set of Aiptasia used to produce Supplementary Figure S1. Each row depicts a replicate anemone. Floridoside levels from Table S12 were normalized to algal endosymbiont cell counts from Table S9.

Click here to Download Table S13

Biology Open • Supplementary information

References

Gegner, H.M., Ziegler, M., Rädecker, N., Buitrago-López, C., Aranda, M., Voolstra, C.R., 2017. High salinity conveys thermotolerance in the coral model Aiptasia. Biol. Open 6, 1943–1948. https://doi.org/10.1242/bio.028878

Biology Open • Supplementary information
